# Tension Pneumoperitoneum as a Result of Diastatic Perforation

**DOI:** 10.7759/cureus.36010

**Published:** 2023-03-11

**Authors:** Atul Ajith, Snehasis Das, Sagar Prakash, Oseen Shaikh, Uday Kumbhar

**Affiliations:** 1 Surgery, Jawaharlal Institute of Postgraduate Medical Education and Research, Puducherry, IND

**Keywords:** tension pneumoperitoneum, laparotomy, inguinal hernia, diastatic perforation, pneumoperitoneum

## Abstract

Tension pneumoperitoneum is a vapid presentation of pneumoperitoneum, which generally refers to free air in the abdomen and can mimic abdominal compartment syndrome. A diastatic perforation in the abdomen refers to a perforation of the cecum due to a distal obstruction in the colon, manifesting as a closed-loop syndrome. We present a 46-year-old male diagnosed with obstructed left inguinal hernia who underwent hernioplasty. Postoperatively, the patient had progressive abdominal distention and abdominal pain. An abdominal x-ray and computed tomography of the abdomen showed massive air in the abdomen. The patient was diagnosed to have tension pneumoperitoneum. Needle decompression of the abdomen was done, and the patient underwent an emergency laparotomy. Intraoperatively, we found a large cecal perforation and a large amount of pneumoperitoneum. The patient underwent limited resection and ileostomy and ascending mucus fistula. Postoperatively, the patient had an uneventful course and was discharged.

## Introduction

Tension pneumoperitoneum is an uncommon, life-threatening form of abdominal compartment syndrome that generates intra-abdominal pressures high enough to compromise vascular flow and cause systemic homeostatic destabilization [1]. It has been theorized to impede venous outflow by increasing the inferior vena cava impedance and decreasing preload [2]. Diastatic perforation due to the closed-loop obstruction of the colon is very rare. Usually, patients present with abdominal pain and abdominal distention. Clinical examination usually shows a diffusely distended abdomen with a resonant note over the abdomen. Imaging studies like abdominal x-rays and computed tomography (CT) help diagnose the disease. Early needle decompression and laparotomy are usually required. We present a 46-year-old male who underwent hernioplasty and was postoperatively diagnosed with tension pneumoperitoneum due to cecal perforation as a result of closed-loop obstruction of the colon.

## Case presentation

A 46-year-old male presented with abdominal distension, abdominal pain, and obstipation for four days. The patient also gives a history of the irreducibility of the left inguinal hernia for four days, which was present for the last 10 years. The patient did not have similar complaints in the past. The patient was conscious and oriented, with a pulse rate of 106 beats per minute and blood pressure of 140/90 mm of Hg. Abdominal examination showed a distended abdomen without any tenderness. Bowel loops were palpable. Left inguinal examination showed the presence of an irreducible left inguinal hernia without significant local tenderness.

**Figure 1:**
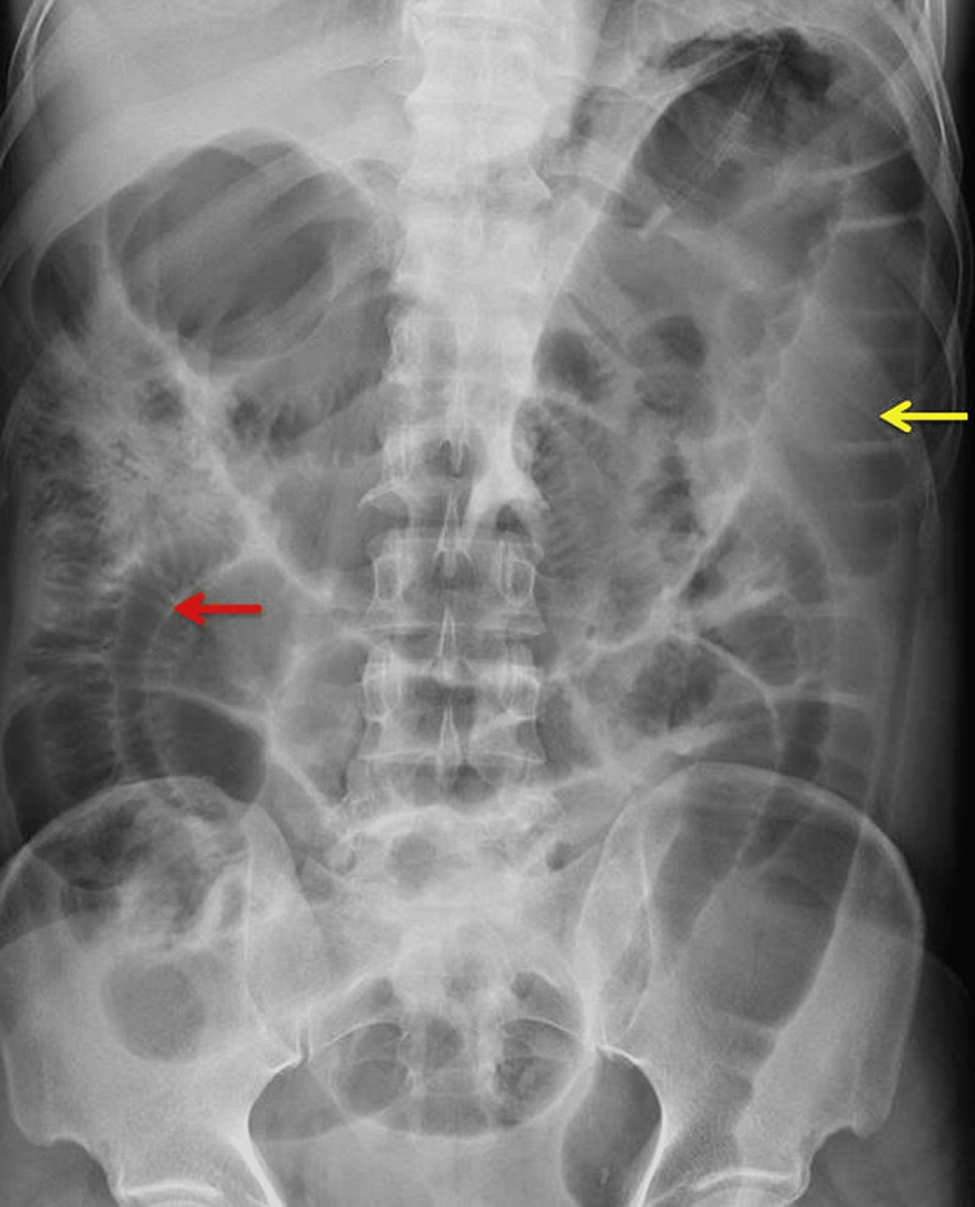
X-ray of the abdomen showing a dilated large bowel loop (yellow arrow) and small bowel loops (red arrow).

All routine blood investigations, including renal function tests and renal function tests, were done and were normal except for the mild leucocytosis. Abdominal x-ray showed significantly dilated large and small bowel loops (Figure 1).

The patient was diagnosed with obstructed inguinal hernia and was taken up for immediate surgical exploration. Inguinal exploration was done, and it was found to contain a sigmoid colon. There was no evidence of strangulation of the bowel. Hence, the bowel was reduced, and a left inguinal hernioplasty was done.

**Figure 2:**
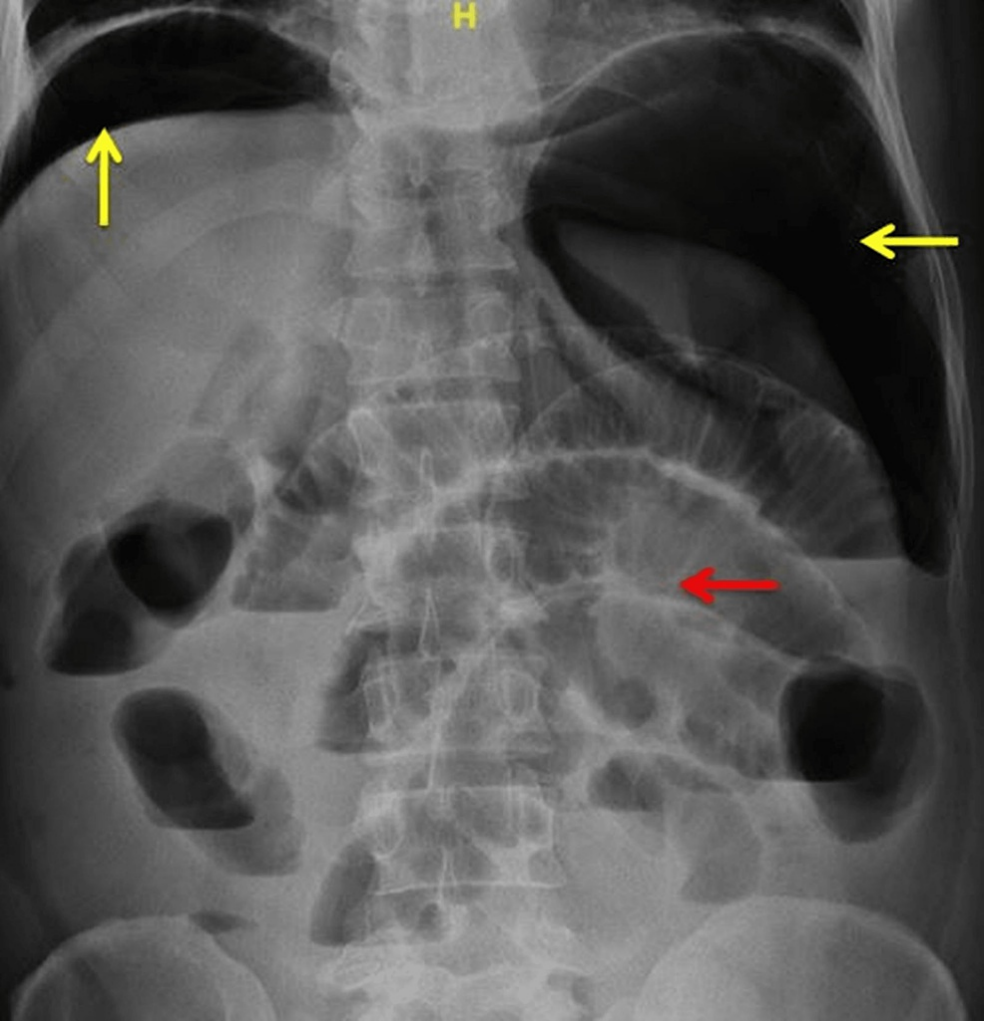
X-ray of the abdomen showing massive pneumoperitoneum (yellow arrows) and dilated small bowel loops with valvulae conniventes (red arrow).

The patient complained of abdominal pain and progressive distension on a second postoperative day. The patient had multiple fever spikes. There was no history of decreased urine output or dyspnea. The patient had a pulse rate of 120 beats/min, a respiratory rate of 20 cycles per minute, and a blood pressure of 100/80 mm of Hg. Abdominal examination showed a distended and diffusely tender abdomen with rigidity. Abdominal x-rays showed massive air under the diaphragm (Figure 2).

**Figure 3:**
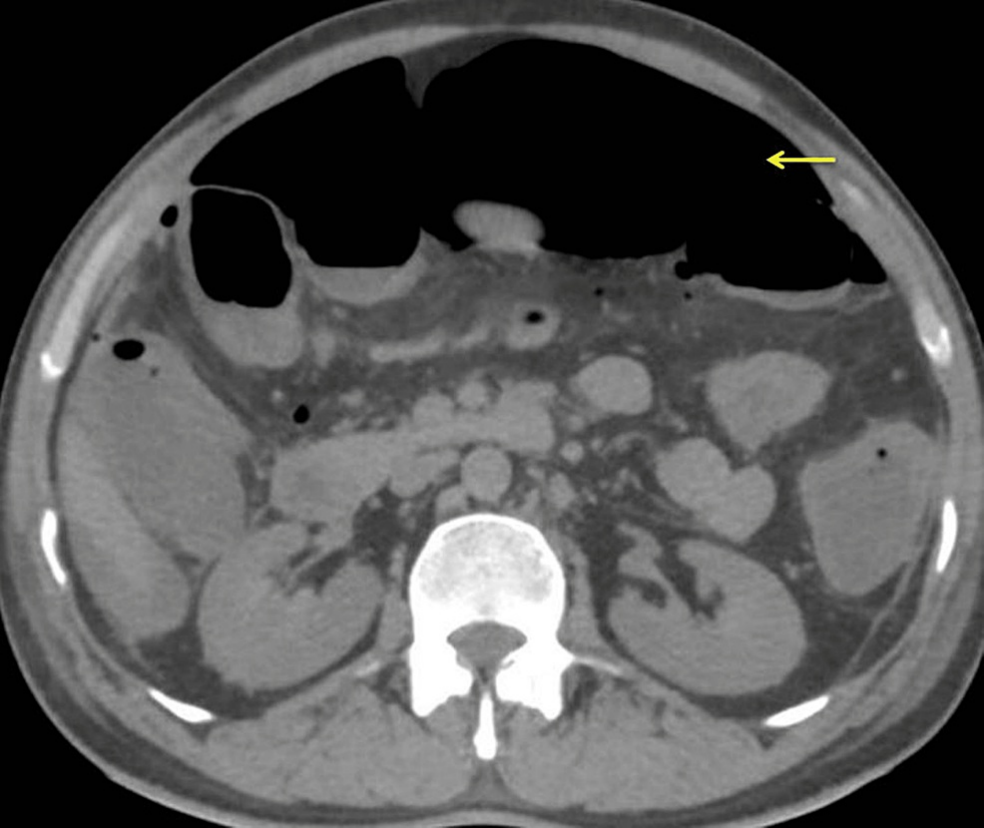
Computed tomography (axial view) showing massive pneumoperitoneum (yellow arrow).

CT of the abdomen showed a massive pneumoperitoneum with significant fat stranding along the right iliac fossa (Figure 3).

**Figure 4:**
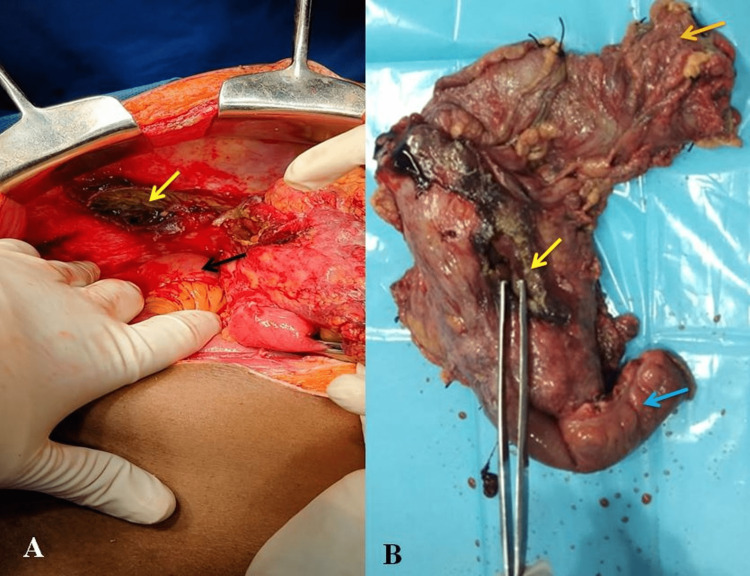
Intraoperative image showing A: Cecal perforation (yellow arrow), and B: Limited resection specimen showing cecum (upper orange arrow), ileocecal junction, and terminal ileum (blue arrow) with a cecal perforation show toothed forceps (middle yellow arrow).

The patient was taken up for immediate exploratory laparotomy. Intraoperatively, we found 200 ml fecopurulent fluid and a 3 cm x 2 cm perforation in the posterior wall of the cecum with necrotic margins with inflamed cecum and distal ileum. Limited resection of the cecum and distal ileum with covering ileostomy and distal ascending colon mucus fistula was done (Figure 4).

The postoperative period was uneventful, and he was discharged after one week.

## Discussion

Perforation of an over-distended, disease-free cecum due to an obstructing lesion in the distal colon is called diastatic perforation. The pathophysiology associated with this phenomenon obeys the Laplace law, which causes the circumference of the gut to increase by three times in comparison to the diameter [3]. This leads to rapid stretching and, along with a competent ileocecal valve, to a perforation. Cecum is the most common site of the perforations as it is the area of maximum outstretch whenever there is increasing pressure in the colon [4].

Regarding pathophysiology, diastatic perforations are theorized to occur by two main mechanisms. Malignant causes lead to direct perforation resulting from tumor necrosis, and benign ones usually form a closed-loop obstruction from a competent ileocecal valve. Cecal perforations are primarily found on the anterior longitudinal axis with sharp uninflamed margins, with other common sites being hepatic flexure, splenic flexure, and the descending colon [5]. Clinically, a prolonged intraluminal centrifugal pressure of 10 cm of water causes mucosal petechial hemorrhages, 20 cm of water causes necrosis and gangrene within 32 hours, and 40 cm of water within 24 h [5]. In our case, the intrabdominal pressure (IAP) stood at a clinical peak of 32 cm of water (measured by measuring bladder pressure through a transurethral foley catheter connected to a pressure transducer), which took at least one or two days to manifest, leading to a delayed second hit phenomenon.

Most cases of such colorectal perforations usually present with classical clinical signs with radiological imaging showing characteristically air under the diaphragm. In suspicious sealed-off perforations, CT abdomen will demonstrate collection, significant fat stranding, or mass formation. Symptoms and signs are outright, which makes the diagnosis evident and easy to tackle. However, in our case, the developing symptoms of closed-loop syndrome from the obstructed hernia and the initial absence of a florid picture of perforation led to a delayed diagnosis. Risk factors heralding the progression from ischemia to a frank perforation, as would have been in our case, depending on a cecal diameter of more than 9 cm, a long-standing dilatation, and an intraluminal pressure of more than 80 mm of Hg tethered by a predisposing closed loop syndrome [5].

Tension pneumoperitoneum is a rare form of abdominal compartment syndrome. It occurs due to the entrapment of a large amount of intraperitoneal air within the abdomen, which increases abdominal pressure. Patients may develop decreased venous return, tachypnoea, bradycardia, decreased urine output, and mesenteric ischemia. Tension pneumoperitoneum as presenting symptom of a diastatic perforation has never been reported in medical literature as per our research. In contrast, most have been seen to be secondary to hollow viscus perforation. Usually, with frank peritonitis and shock-like features, the patient's course is too short for free air to collect inside the abdomen and create significant tension. The most commonly reported causes of tension pneumoperitoneum include complications from gastrointestinal endoscopies, trauma, improper intubation, and scuba diving [6]. In addition to a rare case of tension pneumoperitoneum following an appendicular perforation, benign colonic perforations causing such phenomena have seldom been reported [7].

Patients usually present with an overtly distended abdomen in refractory shock due to cardiorespiratory collapse, florid acute kidney injury, hypotension, and tachycardia. Prompt diagnosis in the form of an abdominal x-ray showing the saddle sign or centralization of the bowel, or in cases of doubt, contrast-enhanced CT helps clinch the diagnosis. In our case, an abdominal x-ray and a plain abdomen CT sufficed in establishing the diagnosis.

Tension pneumoperitoneum is a surgical emergency with emergency laparotomy as the best and definitive treatment modality. If the patient is too unstable for the definitive procedure or anesthetic induction, temporary measures with needle decompression or intra-abdominal drain placement can help mitigate the hemodynamic instability and ventilatory impedance. However, it is also associated with its own risk as a stealing phenomenon [8]. In our case, the patient was taken up for immediate laparotomy, which showed cecal perforation. The patient underwent limited resection and ileostomy with a distal mucus fistula.

## Conclusions

Although reported, the combination of tension pneumoperitoneum and a hollow viscus perforation is a rare presentation due to florid clinical signs before its development. In addition, tension pneumoperitoneum in a case of diastatic perforation was never reported, which makes the diagnosis difficult. As tension pneumoperitoneum has lethal outcomes, clinicians should be aware of such scenarios. Patients who have been diagnosed to have tension pneumoperitoneum need urgent surgical intervention. 
